# Silica as a Matrix for Encapsulating Proteins: Surface Effects on Protein Structure Assessed by Circular Dichroism Spectroscopy

**DOI:** 10.3390/jfb3030514

**Published:** 2012-08-02

**Authors:** Phillip J. Calabretta, Mitchell C. Chancellor, Carlos Torres, Gary R. Abel, Clayton Niehaus, Nathan J. Birtwhistle, Nada M. Khouderchah, Genet H. Zemede, Daryl K. Eggers

**Affiliations:** Department of Chemistry, San José State University, San José, CA 95192-0101, USA; Email: philcalabretta@yahoo.com (P.J.C.); mitch.chancellor@gmail.com (M.C.C.); carlitos408@gmail.com (C.T.); gary.abel@yahoo.com (G.R.A.); claytonniehaus@sbcglobal.net (C.N.); nathan.birtwhistle@sjsu.edu (N.J.B.); caroosa@yahoo.com (N.M.K.); genet_zemede@affymax.com (G.H.Z.)

**Keywords:** biocompatibility, ellipticity, ormosil, protein folding, sol-gel technique

## Abstract

The encapsulation of biomolecules in solid materials that retain the native properties of the molecule is a desired feature for the development of biosensors and biocatalysts. In the current study, protein entrapment in silica-based materials is explored using the sol-gel technique. This work surveys the effects of silica confinement on the structure of several model polypeptides, including apomyoglobin, copper-zinc superoxide dismutase, polyglutamine, polylysine, and type I antifreeze protein. Changes in the secondary structure of each protein following encapsulation are monitored by circular dichroism spectroscopy. In many cases, silica confinement reduces the fraction of properly-folded protein relative to solution, but addition of a secondary solute or modification of the silica surface leads to an increase in structure. Refinement of the glass surface by addition of a monosubstituted alkoxysilane during sol-gel processing is shown to be a valuable tool for testing the effects of surface chemistry on protein structure. Because silica entrapment prevents protein aggregation by isolating individual protein molecules in the pores of the glass material, one may monitor aggregation-prone polypeptides under solvent conditions that are prohibited in solution, as demonstrated with polyglutamine and a disease-related variant of superoxide dismutase.

## 1. Introduction

Although the characterization of biomolecules in dilute solution is useful for determining the properties of the purified molecule, this approach fails to account for excluded volume and hydration effects that occur in concentrated, non-ideal solutions [1]. The neglect of effects associated with concentrated solutions may be especially misleading for biomolecules because their natural environments teem with a broad range of other solutes [2,3,4,5]. Currently, it is difficult to study the three dimensional structure of biomolecules *in vivo* because most analytical techniques are incapable of distinguishing a specific population of molecules from a large mixture, although in-cell NMR spectroscopy has achieved limited success [6,7]. The development of alternative techniques that mimic the effects of macromolecular crowding *in vitro* may lead to new biomaterials and, at the same time, facilitate our understanding of biochemistry *in vivo*.

Related to the issue of crowding effects, methods have been developed to encapsulate proteins in nanoporous materials. One particular method uses the sol–gel technique to confine protein molecules in inorganic glass matrices under relatively mild conditions of temperature and pH. Sol-gel glasses are formed in a two-step process from metal alkoxide precursors like tetramethoxysilane (TMOS). In the first step, methanol is released by acid hydrolysis of the precursor forming a stable “sol” of Si(OH)_4_. In the second step, the mixture is neutralized with aqueous buffer to initiate a condensation reaction that “gels” into a network of [Si–O–Si] bonds. Sol-gel glasses can be formed in optically transparent shapes, facilitating analysis of the entrapped molecules by many of the same spectroscopic methods used for solutions. In 1992, it was first demonstrated that proper buffering of the sol–gel mixture allows one to encapsulate purified proteins without detectable denaturation [8]. Since this prominent paper, many other proteins have been encapsulated in sol-gel glasses for which protein selection has been motivated primarily by potential applications in biosensor and biocatalyst development [9,10,11,12,13,14]. In general, silica-entrapped proteins are unable to diffuse out of the glass matrix, but water and small solutes may diffuse through the interconnected pores, allowing one to alter the solvent conditions after a suitable equilibration period.

In the current study, TMOS-derived glasses are utilized to examine the effects of crowding and confinement on the structure of model proteins. Changes in protein secondary structure are monitored by circular dichroism (CD) spectroscopy as a function of several variables. Although CD measurements may be considered less sensitive than fluorescence techniques, one may monitor the average global structure of the protein by CD; in the far-UV region, the entire protein serves as the chromophore due to the differential absorbance of left-handed and right-handed circularly polarized light by the peptide units that define the backbone. Thus, if any portion of the encapsulated protein is folded differently than the structure found in solution, no matter where the change occurs within the primary sequence, it should be detectable as a change in ellipticity, the unit of measurement for CD spectroscopy.

One less-recognized benefit of silica entrapment is the ability to perform experiments with aggregation-prone polypeptides in the absence of intermolecular aggregation. If the majority of the polypeptide molecules occupy individual pores as monomers, then the pore walls will prevent any interactions with neighboring polypeptides that lead to aggregation. This attribute of silica entrapment is demonstrated here with two disease-associated polypeptides, polyglutamine and a mutant variant of human Cu-Zn superoxide dismutase. 

## 2. Results and Discussion

### 2.1. Studies with Human Copper-Zinc Superoxide Dismutase

Copper-zinc superoxide dismutase (Cu/Zn-SOD) is an enzyme that protects living cells from oxidative damage by catalyzing the disproportionation of superoxide anion to dioxygen and hydrogen peroxide [15]. The protein consists of two identical subunits, each 153 amino acids in length and each containing one copper and one zinc ion cofactor. The copper ion plays a direct role in the catalytic mechanism, whereas the zinc ion acts primarily as a structural component. Mutations in Cu/Zn-SOD have been identified in a subset of human patients suffering from amyotrophic lateral sclerosis (ALS), also known as Lou Gehrig’s disease. In brief, aggregation of the Cu/Zn-SOD protein is believed to precede the death of motor neurons leading to symptoms of the disease. Because protein aggregation typically involves a misfolding event, characterization of the folding pathway of Cu/Zn-SOD is desirable for understanding the cellular mechanism of the disease. In particular, the identification of a stable, partially-folded intermediate state that leads to aggregation would represent a possible target for drug intervention.

**Figure 1:**
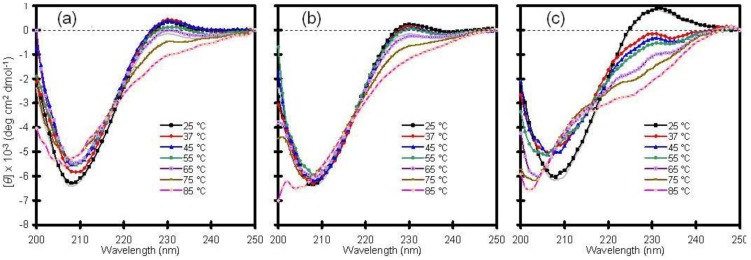
Thermal stability of human copper-zinc superoxide dismutase (Cu/Zn-SOD). (**a**) Wild-type Cu/Zn-SOD in solution; (**b**) Wild-type Cu/Zn-SOD entrapped in silica glass; (**c**) A4V mutant of Cu/Zn-SOD entrapped in silica glass. The vertical axis is molar ellipticity in the traditional units of circular dichroism (CD) spectra. Note that the A4V mutant in panel c displays a significant change in structure at 37 °C and above, whereas the wild-type protein is stable to much higher temperatures.

Because aggregation is disallowed when the protein is entrapped in a silica matrix, the encapsulation technique may provide a means to detect and study folding intermediates that are otherwise difficult or impossible to monitor as free molecules in solution [16]. With this in mind, the thermal stability of Cu/Zn-SOD was examined by heating samples from 25 to 85 °C and by checking the CD profile at discrete temperatures (Figure 1).

Consistent with other reports, wild-type Cu/Zn-SOD exhibited high thermal stability with little change in secondary structure up to 65 °C. The temperature-dependent CD spectra of the wild-type protein in solution were similar to those obtained following encapsulation in a standard silica glass (compare panels a and b of Figure 1). A mutant variant of the enzyme, containing a switch from alanine to valine in the fourth position of the amino acid sequence, behaved much differently. The A4V protein, which is associated with rapid progression of the disease, showed a significant change in secondary structure upon mild heating from 25 to 37 °C, and the fraction of unfolded protein increased with increasing temperature (Figure 1c). This result confirms that the mutant is less stable than wild-type Cu/Zn-SOD and supports the hypothesis that mutant SOD proteins may be more susceptible to aggregation due to a general reduction in conformational stability [17]. The crystal structure of the metal-free A4V mutant indicates that two important loop elements are disordered [18], suggesting that these local regions may initiate the unfolding pathway. The A4V variant has been reported to aggregate spontaneously in solution at 37 °C [19], and, consequently, the results in Figure 1c are difficult to reproduce in solution because the protein precipitates with time, resulting in a weak or misleading signal by CD spectroscopy. Although a stable intermediate state was not identified in this project [16], glass encapsulation appears to be a viable approach for characterizing aggregation-prone polypeptides, as demonstrated here with the A4V mutant of Cu/Zn-SOD.

### 2.2. Xerogel-Encapsulated Type I Antifreeze Protein

Antifreeze proteins, or AFPs, are a group of structurally diverse polypeptides that share the ability to inhibit the growth of ice crystals in certain organisms, including marine fishes and insects [20]. A subclass known as type I AFP, or AFP I, has been isolated from winter flounder. This polypeptide is only 37 amino acids in length and is believed to adopt an extended α-helix conformation when bound to the face of an ice crystal.

**Figure 2:**
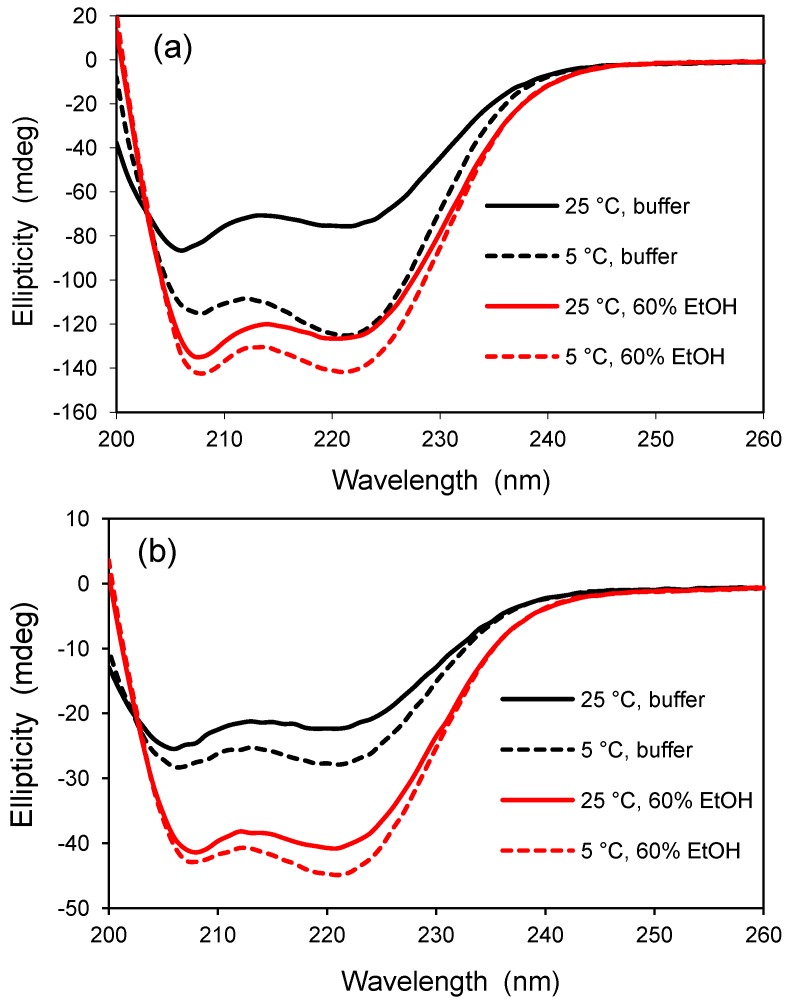
Temperature and solvent dependence of type I antifreeze protein (AFP) before and after encapsulation in silica glass. (**a**) CD analysis of AFP I in solution. AFP concentration is 0.050 mg/mL in a 2-mm pathlength cuvet; (**b**) CD analysis of AFP I encapsulated in a silica xerogel. AFP concentration is approximately 0.084 mg/mL in a 0.75 mm thick glass sample. The CD signal was not converted to molar ellipticity due to uncertainty in the concentration of protein in the xerogel following equilibration. All solvents contain 10 mM potassium phosphate buffer, pH 6.8.

Because AFP I was designed by nature to interact with the periodic placement of oxygen atoms in an ice crystal, one might expect AFP I to bind to the oxygen atoms on the surface of a silica glass, leading to a stable secondary structure. For this reason, AFP I was encapsulated in a standard TMOS-derived glass and examined by CD spectroscopy. Initial studies were disappointing because the CD signal was consistently weak, suggesting a possible problem with protein leaching from the glass material. The leaching issue was confirmed by following the time-dependent loss in fluorescence of a labeled protein [21]. To overcome the loss of protein, subsequent glass samples were allowed to dry and shrink slowly at room temperature, forming a xerogel material. The smaller pore sizes of the xerogel should greatly reduce protein leaching but still lead to an optically transparent material for analysis by CD spectroscopy. Typical results are shown in Figure 2 which compares the secondary structure of AFP I in solution and in the xerogel at two temperatures, 5 and 25 °C, and in two solvents, buffered water and a mixture of 60% v/v ethanol in water. In this case, the CD signal is not given in units of molar ellipticity, but the CD spectra for each panel are compared on the basis of constant protein concentration (see Experimental Section 3.6).

The CD spectra in Figure 2 are consistent for a peptide with a high degree of helicity, as indicated by the characteristic minima near 208 and 222 nm. As expected from other reports, AFP I was found to be more helical at the lower temperature of 5 °C compared to 25 °C, and this observation was evident both in solution and in the xerogel. The effect of temperature on helicity was stronger in solution than detected in the xerogel, presumably due to crowding and confinement effects in the silica matrix. The presence of 60% ethanol increased the ellipticity of the polypeptide under both conditions, revealing that the structure does not reach maximum helicity in water at 5 °C. Alcohols are known to induce helical structure in proteins, and, for a given alcohol concentration, the helical content increases with the alkyl chain length of the alcohol [1]. This study demonstrates that glass encapsulation studies are not limited to proteins larger than 50 amino acid residues if the glass is dried slowly during the aging process to reduce the average pore size. 

### 2.3. Entrapment of Polyglutamine, a Disease-Related Aggregation-prone Peptide

Polyglutamine diseases, which include Huntington’s disease, are caused by the insertion of an expanded trinucleotide repeat sequence in specific genes. The codon for glutamine, CAG, is repeated sequentially in the DNA sequence leading to a mutated protein [22]. Once the expansion exceeds a critical length of 35–45 repeats, the affected peptide is prone to forming stable, irreversible aggregates known as inclusion bodies. The molecular mechanism of the disease is unclear, but one hypothesis is that soluble oligomers of the mutant protein, formed by interactions between their polyglutamine regions, are responsible for the gain-in-toxic function that leads to neurodegeneration [22]. 

Model peptides containing a string of continuous glutamine residues of varying length have been synthesized for solution studies, and numerous results suggest that polyglutamine sequences adopt a random coil structure as soluble monomers but eventually form small soluble aggregates with β-strand structure [23,24]. The monomer can be induced to change from a random conformation to a helix by addition of alcohols including trifluoroethanol [25].

**Figure 3:**
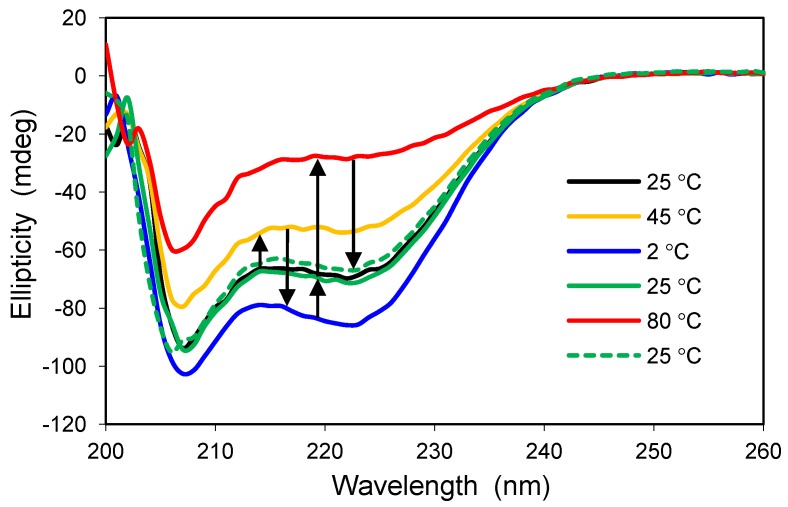
Thermal response of polyglutamine structure when confined to the pores of a silica xerogel modified with 10% fluoropropyl groups. The same glass sample was sequentially heated or cooled as indicated by the arrows, moving from left to right. The glass was equilibrated in buffered water, pH 6.8.

Because polyglutamine peptides are prone to aggregation over time, encapsulation in silica glass should allow one to study the folding properties of the monomer under conditions where aggregation is minimized or prohibited. A peptide containing 15 glutamine repeats, capped with two aspartate residues on the N-terminus and two lysine residues on the C-terminus to enhance solubility, was encapsulated in an organically-modified silica glass made with 90% TMOS and 10% trifluoropropyl-trimethoxysilane. The fluorocarbon modifying reagent has been shown to induce helical structure in another protein and, therefore, was employed to enhance the solubility of the polyglutamine peptide during sol–gel processing [26]. Because of the relatively small size of the peptide, the glass was allowed to dry slowly, similar to the AFP I protocol, in order to form a xerogel and minimize leaching. The temperature-dependent structure of encapsulated polyglutamine is given in Figure 3.

Encapsulated polyglutamine yields a helical conformation in the 10% trifluoropropyl glass at 25 °C, and the structure changes reversibly by altering the temperature; increases in temperature reduce the fraction of helical structure, and decreases in temperature enhance the helical content (Figure 3). The reversibility of conformations and the lack of ellipticity corresponding to β-structure in the CD profile suggest that the peptide was successfully entrapped as a monomer. Studying the temperature-dependent behavior of polyglutamine in solution is not possible at such extreme temperatures due to aggregation. The enhanced helical content of polyglutamine upon lowering the temperature to 2 °C indicates that this property is not unique to antifreeze proteins. The gain in helicity may reflect a preference for intermolecular H-bonds in the peptide backbone over peptide-water H-bonds as the entropy penalty diminishes with decreasing temperature. Similar encapsulation experiments have been carried out with an Aβ peptide associated with Alzheimer’s disease [27].

### 2.4. Effects of Hydrophilic Surface Modifications on Apomyoglobin Structure

Apomyoglobin (apoMb) refers to the myoglobin protein molecule after removal of the iron heme cofactor that confers its ability to bind oxygen. Previous encapsulation studies with apoMb indicate that this marginally-stable helical protein is an excellent reporter of changes in the aqueous environment. ApoMb is largely unfolded in a silica glass (100% TMOS) but responds favorably to high molar concentrations of potassium phosphate (KPhos) and other secondary solutes [1,28]. Recent studies indicate that glass-encapsulated apoMb becomes more helical as the silica surface becomes more hydrophobic due to the incorporation of monosubstituted alkyl modifying reagents [26,29].

**Figure 4:**
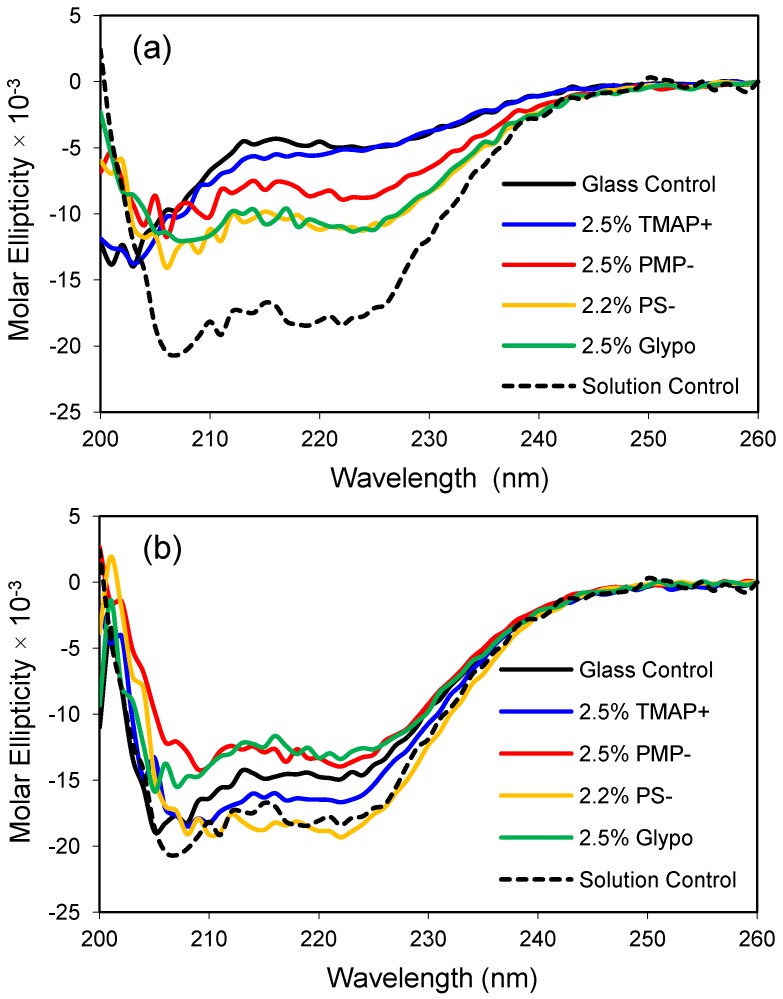
Apomyoglobin encapsulated in polar organically-modified glasses. (**a**) CD spectra after equilibration in low phosphate buffer (10 mM, pH 6.8); (**b**) CD spectra after equilibration in high phosphate buffer (1.0 M, pH 6.8). The dashed line represents the CD profile of the native protein in solution, and the control is an unmodified glass made from 100% tetramethoxysilane (TMOS).

In the current study, modifying agents containing polar functional groups are examined for their effects on the secondary structure of apoMb. More specifically, small amounts (2.0%–2.5% on a molar basis) of the following reagents were mixed with TMOS to formulate the hydrophilic glasses: trimethylammoniumpropyl-trimethoxysilane (TMAP^+^), trihydroxysilyl-propylmethylphosphonate (PMP^−^), trihydroxysilyl-propylsulfonic acid (PS^−^), and glycidoxypropyl-trimethoxysilane (Glypo). The effect of these glass modifiers on the structure of apoMb are summarized in Figure 4.

ApoMb responds favorably to modest changes in glass composition, as demonstrated by a gain in the molar ellipticity of the protein in the modified glasses relative to the control glass made from 100% TMOS (Figure 4). Presumably, the polar functional groups are found on the surface of the pore walls and interact directly with the protein or influence the properties of the confined water. Interestingly, the cationic TMAP^+^ glass had the weakest effect in low salt buffer but provided one of the most native-like CD profiles in the higher 1.0 M phosphate buffer (compare panels a and b). For this set of experiments, the anionic sulfonate glass (PS^−^) resulted in the one of the most helical protein conformations at both low and high salt conditions. A future goal of these studies is to obtain a native-like solution structure for encapsulated apoMb without the addition of secondary solutes like phosphate. Glass compositions that provide a native-like CD profile for apoMb are expected to provide a biocompatible environment for encapsulation of other proteins, as well.

### 2.5. Confinement Effects on Polylysine

**Figure 5:**
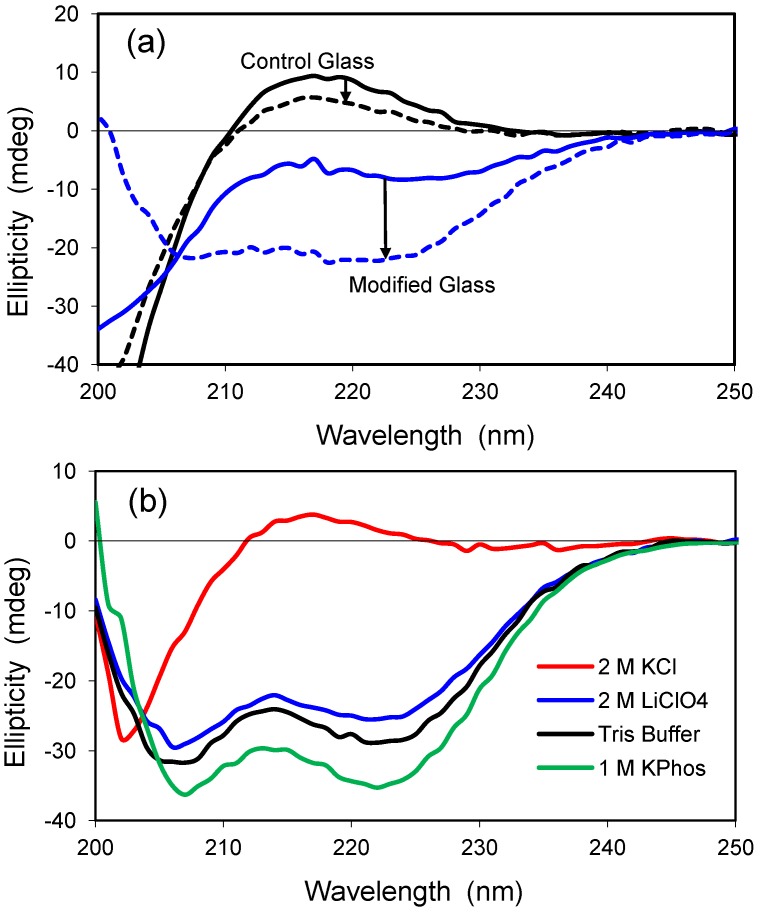
(**a**) CD spectra of polylysine in two glasses. Solid lines represent standard buffer condition (pH 7.4), whereas the dashed lines indicate addition of 2.0 M LiClO_4_. The spectrum in the control glass with no salt is similar to that obtained in solution at neutral pH. The control glass is 100% TMOS; the modified glass is a combination of 5% trimethylammoniumpropyl-trimethoxysilane (TMAP^+^) and 5%trihydroxysilyl-propylmethylphosphonate (PMP^−^); (**b**) CD spectra of polylysine in 2.5% Glypo glass equilibrated in four different solutions at pH 7.4, as indicated in the key.

Polylysine is a positively-charged model peptide that can adopt the three most common secondary structures found in proteins, α-helix, β-sheet, and random coil [30]. At neutral pH, polylysine is random in solution but becomes helical as the pH is increased toward the pK value of lysine’s ε-amino sidechain (pK ≈ 10.5). Similar to solution, polylysine has a highly random structure in the control glass at neutral pH, as exemplified by the positive ellipticity and peak near 218 nm (Figure 5a). The random conformation changes only slightly in the presence of 2.0 M LiClO_4_, a salt known to induce helical structure for polylysine in solution [31]. Encapsulation within a modified glass having both cationic and anionic groups leads to a slight increase in ellipticity, and the polypeptide structure adopts a helical conformation upon addition of 2.0 M LiClO_4_. Thus, the polypeptide behaves more like it would in solution when it is confined to the hydrophilic, organically-modified silica glass. 

Polylysine was examined further after encapsulation in a hydrophilic, uncharged glass of 2.5% Glypo (Figure 5b). Polylysine remained in a random coil conformation when 2.0 M KCl was present, but a structure of high helicity was detected in solutions of 10 mM Tris buffer, 1.0 M KPhos, and 2.0 M LiClO_4_. These glass-encapsulated spectra are remarkable because Tris buffer and 1.0 M KPhos do not significantly alter the random conformation of polylysine in solution. Although the Glypo-modified glass does not provide any charged groups on the silica surface to interact electrostatically with the charges of polylysine, it is possible that the epoxide ring of the glycidoxyalkyl group reacts with the ε-amino groups of polylysine, thereby linking the protein covalently to the silica surface. Experiments using mass spectrometry to confirm the reactivity of the Glypo reagent under solvent conditions that mimic the sol-gel process were inconclusive (data not shown).

## 3. Experimental Section

### 3.1. Materials

Tetramethoxysilane was obtained from Sigma-Aldrich (St. Louis, MO, USA), and all silica-based modifying reagents were purchased from Gelest (Morrisville, PA, USA). Cu/Zn-SOD proteins were a gift from U. Texas Health Science Center at San Antonio (P.J. Hart). Antifreeze protein was obtained from A/F Protein (Waltham, MA, USA), and the polyglutamine peptide was synthesized by AnaSpec (San José, CA, USA). Apomyoglobin was prepared from horse heart myoglobin [32] purchased from Sigma-Aldrich, and polylysine was also obtained from Sigma-Aldrich. Plastic cassettes for casting glass sheets were obtained from Invitrogen (Carlsbad, CA, USA). Buffers, salts, and other chemical reagents were purchased from Fisher Scientific.

### 3.2. Standard Glass Formation

The standard sol was made using TMOS, dilute HCl, and water; the recipe calls for 1.0 mL of TMOS, 30 μL of 0.040 N HCl, and 214 μL of water in a 15-mL plastic conical tube which was sonicated in an ice-water bath for 20–30 minutes [1]. Typically, 2 or 3 tubes of sol were made and combined for each glass sheet. Protein solutions of desired concentration were prepared in 10 mM KPhos buffer, pH 6.8, unless stated otherwise. The volume ratio of sol to aqueous protein solution was maintained at 2:3. The sol and aqueous volumes were gently mixed and immediately transferred to a plastic cassette with plates of 1-mm spacing. The glass was allowed to solidify at ambient temperature, covered with parafilm to reduce solvent evaporation, and moved to a refrigerator. After 12–24 hours, the glass was layered with water and allowed to age for approximately two weeks. After the wet-aging period, the cassette was carefully pried open, and the resulting sheet of silica was cut into wafers approximately 0.8 cm × 2 cm in size. The rectangular glass wafers were transferred into 3 mL of the desired solvent and allowed to equilibrate with the solution for a minimum of 12 hours prior to analysis by CD spectroscopy.

### 3.3. Xerogels for AFP Entrapment

The steps in making a xerogel for AFP I encapsulation are the same as that of the standard glass except for the aging process and choice of cassette. A plastic cassette with 1.5 mm spacing was employed to account for shrinkage, yielding a final xerogel of approximately 0.75 mm thickness. Upon solidification of the glass, 13–15 holes were made in the parafilm with a hypodermic syringe needle to allow slow evaporation of the solvent, primarily methanol. After 24 hours, the parafilm was removed and the sample was allowed to dry for a minimum of two weeks at room temperature. To prepare xerogel samples for CD analysis, the dry silica wafers were stored in an airtight container with a wet tissue to slowly rehydrate the silica in a humid environment. Direct immersion of xerogel samples in water without the slow rehydration step resulted in severe cracking and prevented further analysis. The characteristic shrinkage of a xerogel results in a higher effective protein concentration which must be taken into account if one converts the raw data to molar ellipticity.

### 3.4. Polyglutamine Encapsulation in Fluoropropyl-modified Xerogel

A protocol from Chen and Wetzel was adapted for initial solubilization of the polyglutamine peptide [33]. A few milligrams of peptide were brought up in 1.5 mL of a 50:50 mixture of trifluoroacetic acid and trifluoroethanol. Nitrogen gas was bubbled through the sample at a rate of a few bubbles per second until the sample appeared dry. The pre-treated peptide was resuspended in 1.2 mL of 1.00 mM TFA in water and allowed to clarify by resting undisturbed for approximately 1 hour. Glasses were made by the standard protocol except the sol contained 10% trifluoropropyl-trimethoxysilane and 90% TMOS on a molar basis. After aging two weeks in contact with water, wafers were cut, placed in a plastic container, and covered with parafilm to make an air-tight seal. Next a few pinholes were made in the parafilm, and the glass samples were allowed to age for a minimum of two more weeks at 4 °C, at which point the samples appeared to stop shrinking. Similar to AFP xerogel samples, the polyglutamine wafers were slowly rehydrated on a timescale of days.

### 3.5. Hydrophilic, Organically-modified Glasses

The TMAP^+^ and Glypo glasses were made by adding the desired percentage of the modifying reagent to a volume of TMOS such that the total amount of silane in the sol preparation is identical to that in the standard glass preparation. The Glypo sol was prepared by co-hydrolysis with TMOS in the same tube during the sonication step, whereas the TMAP^+^ sol was hydrolyzed separately from TMOS before mixing and addition of the aqueous protein. 

In the cases of PS^−^ and PMP^−^ glasses, the modifying reagents were supplied in the silanol form so no hydrolysis was required. However, the PS^−^ reagent is highly acidic and the PMP^−^ reagent is highly basic, mandating a neutralization step with strong base or acid, respectively, prior to mixing with the TMOS sol and addition of the aqueous protein solution. Best results were obtained by keeping all solutions on ice and by maintaining the cassette near 4 °C during the casting and aging steps.

### 3.6. Circular Dichroism Spectroscopy

Changes in protein structure were monitored with an Aviv Model 215 circular dichroism spectrometer equipped with a Peltier-type thermoelectric cell holder. Glass samples were placed in a 2-mm pathlength quartz cuvet filled with the corresponding solvent during analysis. A buffer sample containing no protein was subtracted from all spectra to account for any background signal. For those figures in which the ellipticity is reported in units of millidegrees (mdeg), the multiple CD profiles reflect the same concentration of protein in solution or correspond to glass samples made with the same amount of protein. Thus, any error in calculation of the absolute protein concentration is controlled internally and has no effect on the interpretation of experimental results.

## 4. Conclusions

Circular dichroism spectroscopy is a powerful tool for monitoring changes in the structure of encapsulated biomolecules prepared by the sol-gel technique. If the glass material can be prepared with the property of optical transparency, one may study the effects of confinement, solute addition, and surface chemistry on the secondary structure of entrapped proteins. Glass compositions that result in native-like protein structure, as defined by the protein conformation in solution, are viewed as the most biocompatible materials for protein applications.

In the current work, another benefit of glass entrapment was demonstrated, the ability to study aggregation-prone polypeptides by spectroscopic techniques under conditions where the protein is known to aggregate in solution. This ability may be especially useful in the characterization of disease-related proteins, such as the Cu/Zn-SOD mutant and polyglutamine peptide examined here. In the case of smaller peptides, the silica matrix can be slowly dried to form a xerogel with smaller pore sizes to reduce protein leaching, as accomplished with AFP I and polyglutamine. Many silica modifying reagents are commercially available for altering the chemistry of the glass surface and for examining the response of encapsulated proteins. Modest increases in hydrophilicity had a significant effect on the conformations of silica-entrapped apomyoglobin and polylysine.
